# Data representations and -analyses of binary diary data in pursuit of stratifying children based on common childhood illnesses

**DOI:** 10.1371/journal.pone.0207177

**Published:** 2018-11-29

**Authors:** Johan de Rooi, Sarah K. Nørgaard, Morten A. Rasmussen, Klaus Bønnelykke, Hans Bisgaard, Age K. Smilde

**Affiliations:** 1 Biosystems Data Analysis Group, Swammerdam Institute for Life Sciences, University of Amsterdam, Amsterdam, The Netherlands; 2 COPSAC, Copenhagen Prospective Studies on Asthma in Childhood, Herlev and Gentofte Hospital, University of Copenhagen, Copenhagen, Denmark; 3 Department of Food Science, Faculty of Science, University of Copenhagen, Copenhagen, Denmark; TNO, NETHERLANDS

## Abstract

In this article we analyse diary reports concerning childhood symptoms of illness, these data are part of a larger study with other types of measurements on childhood asthma. The children are followed for three years and the diaries are updated, by the parents, on a daily basis. Here we focus on the methodological implications of analysing such data. We investigate two ways of representing the data and explore which tools are applicable given both representations. The first representation relies on proper alignment and point by point comparison of the signals. The second approach takes into account combinations of symptoms on a day by day basis and boils down to the analysis of counts. In the present case both methods are well applicable. However, more generally, when symptom episodes are occurring more at random locations in time, a point by point comparison becomes less applicable and shape based approaches will fail to come up with satisfactory results. In such cases, pattern based methods will be of much greater use. The pattern based representation focuses on reoccurring patterns and ignores ordering in time. With this representation we stratify the data on the level of years, so that possibly yearly differences can still be detected.

## Introduction

Medical research often revolves around experiments or measurements which can only be conducted in the clinic or performed at the lab. Logistics, costs and man power often limits the numbers of visits of participants to the clinic. This can be a serious bottleneck in cases where one is interested in detecting more subtle variations over time. A partial solution is found in augmenting the clinical investigations with other types of data that are more easily collected. In this article we analyse diary reports concerning childhood symptoms of illness, these data are part of a larger study on childhood asthma [1]. The children are followed for three years and the diaries are updated, by the parents using pen and paper, on a daily basis. Comparable studies in the literature are for instance [2] and [3].

The aim of this diary study at large is to combine these data with other types of data in order to come to a better understanding of childhood asthma. On a lower level it is the idea to cluster the symptoms as recorded in the diaries and use these for better phenotyping in a next step. Because of the unusual properties of the diary data, which will be discussed below, we devote this paper to the methodological implications of analysing the diaries. A more thorough discussion of the background of the data and a clinical interpretation of the findings will be presented elsewhere.

All variables in the diaries are measured on a binary scale. Analysing such data raises two major methodological issues. The first deals with what kind of representation is suitable for these type of data and also facilitates further analysis. At a general level we take two approaches, both will be briefly introduced here, but are extensively discussed in the next section. The first approach is based on aligning and summarizing the diaries in such a way that they can be compared in a point by point fashion (we call this a shape based representation). The second approach is inspired on applications in text mining and treats every diary as a collection of patterns (which is called pattern based representation in the following).

The second issue we address follows from the first and concerns the actual (statistical) tools that are most applicable to analyse the (restructured) data. One of the choices here is whether the data should be analysed as having a three-way structure, or bring it back to a two way structure. Because the aim of this paper is exploratory, only unsupervised methods are used, all are introduced in the methods section.

The characteristics of the collected data and the properties of the missing values in the diaries are discussed in the next section. The methods section is followed by a discussion of the results. We close with a discussion of the relative merits and demerits of the different methods and draw some conclusions with respect to the approaches taken.

## Materials and methods

### Data representation

The study was conducted in accordance with the guiding principles of the Declaration of Helsinki and was approved by the Local Ethics Committee (COPSAC2010: H-B-2008-093) and the Danish Data Protection Agency (2015-41-3696). All parents gave written informed consent before enrolment. The dataset consists of 700 diary reports concerning ten common childhood symptoms of illness. This research is designed and conducted by COPSAC (Copenhagen Prospective Studies on Asthma in Childhood) [1]. The first children entered the study in 2009, data collection ended in 2014. The aim was to monitor each child for three years and children could enter the study at any time of the year.

**Fig 1:**
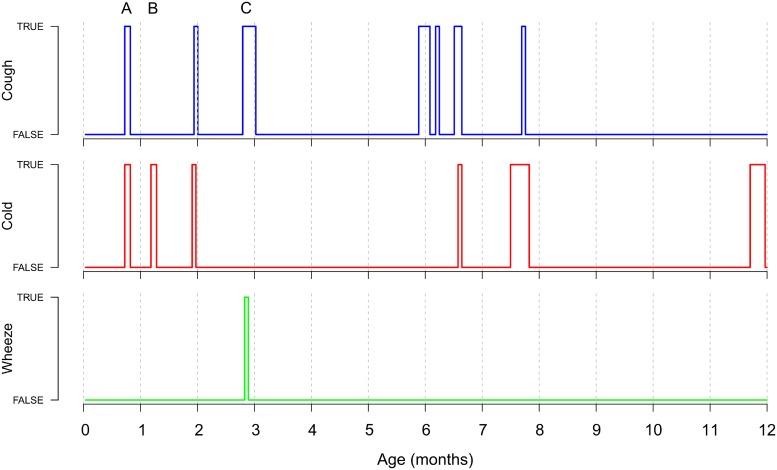
The observed binary scores for three symptoms measured over the first year of the life of one child. Highlighted by the grey bars are three (arbitrary) periods. One with both cold and cough symptoms (A), a second with only cold symptoms (B), the third one marks a number of days with cough and wheezing symptoms (C).

For each child or diary we have a matrix X, consisting of *M* columns for the symptoms, one column registering the date and T rows for the total number of registrations. One column from such a matrix can be thought of as a binary signal over time. Every day a particular symptom occurs, the signals equals one. If the symptom is absent the signal is zero. Three examples of such a signal, stretching over one year, are depicted in Fig 1.

The choice for binary scales facilitates filling in the diaries. Especially in the case of young children, it is more easy to decide on the actual presence or absence of a symptom compared to scoring the severity of the symptom on e.g. a Likert scale. At the same time this also restricts the scope of the analysis. Comparisons of the symptom burden between children are made in terms of the (co)occurrence of symptoms and not their severity.

To compare all diaries they need to be combined, however the different entrance dates of the children complicates a simple merger into one data file. In addition, not all diaries have data for the complete three years. More details on the incomplete diaries are given in the next section. Thereafter we discuss two strategies to align the different files in order to make them comparable.

#### Incomplete diaries

**Fig 2:**
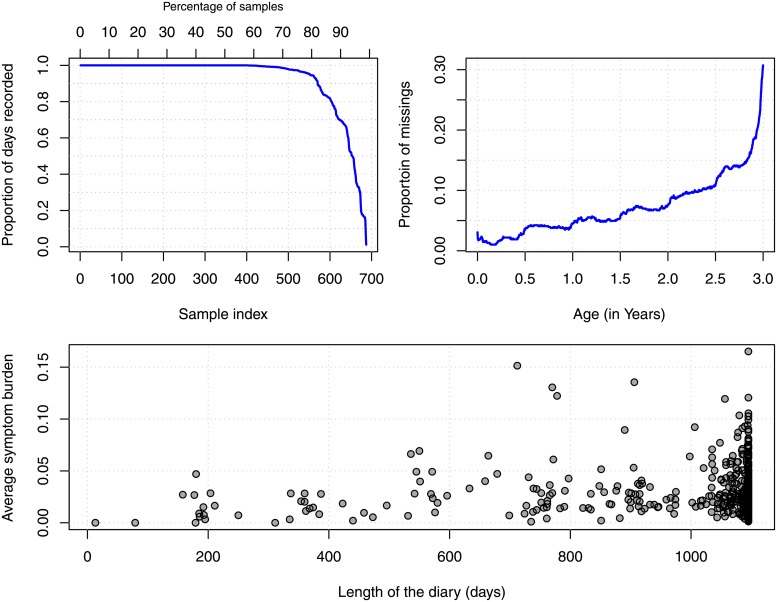
Inspecting the missing values in the diaries. The left upper panel shows that almost 60 percent of the diaries are complete. The missings are clearly related to the time the child and parents are enrolled in the study, as can be seen in the right top panel. The bottom panel shows that there is no relation between the symptom burden and drop-out.

From the 700 children, 403 have a diary that is filled-in for the total of three years, 570 diaries contain information stretching 1000 days or more. The number of recorded days for all diaries is visualized in the upper left panel of Fig 2. Missing values sometimes occur for a short period somewhere during the three years of study. In most cases the missing records occur towards the end of the diary and are often concentrated in one or a few episodes. The proportion of missings, given age (ignoring different entrance dates), is depicted in the right top panel of Fig 2. For example, when the children have reached the age of 2.5 years, 10 percent of the data are missing at that point. Interesting are the small upward jumps at each half year, this suggests parents most often quit the study at well defined in time, e.g. when the child is one or two years of age or after a (planned) visit to the clinic.

It might be that the actual drop out in the study is related to the symptom burden. This is more formally known as nonignorable nonresponse or missing not at random [4]. It could be that parents find it to difficult to keep the diary while having a child with many complaints. Or, on the contrary, when the child shows no signs of asthma, they think it is needless to fill in the diary. The lower panel in Fig 2 plots the total length of the diaries (number of participated days) against the average symptom burden calculated over all symptoms. The data shows some increased variance in the symptom burden towards the end of the diaries, but the relation between drop out and the average burden up to that point seems very weak. In summary it seems that, apart from the strong relation with time, drop-out is not related to important variables in the study.

**Fig 3:**
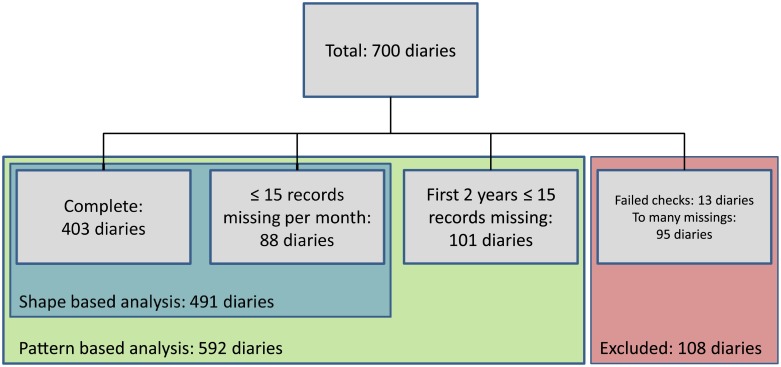
A block diagram showing the number of samples included in both representations.

Fig 3 shows how many diaries are used, depending on the type of data representation. The shape based approach relies on monthly averages, as will be explained in the next section. The advantage of taking means is that, in addition to the 403 complete diaries, potential many more can be included for analysis. To include more samples, diaries are allowed to have up to 15 days of missings per month. This adds 88 diaries and results in a total of 491 diaries, in Fig 3 depicted in the blue rectangle.

In the pattern based representation the data are stratified over years. Meaning that every diary has one row for every year of data included, which results in three rows for each of the just described 491 diaries. On top of these, we included an additional 101 diaries. These diaries have sufficient data in the first two years, but too many missings in the third. As such they all have two rows of data in the matrix. The total set of samples for the pattern based representation is depicted in the green rectangle of Fig 3.

#### Shape based comparisons

The shape based representation follows the traditional way of thinking when comparing signals or repeated measurements. This amounts to point by point comparisons of all the samples. It essentially means we are comparing differences in shape and magnitude of the signals over the time dimension.

Very important in this approach is that all samples are properly aligned. Yet, aligning the diary data, as measured in the current setting, is non trivial. We distinguish interfering effects at three different levels:

- Age
- Season
- Year

Children entered the study at any date, roughly between early 2009 and the spring of 2011. As such, aligning the children based on their age might be the most simple solution. This corrects for effects like maturation of the immune system and admission to daycare. However, next to these influences, there are very strong seasonal effects. One example is the common cold, which is much more abundant in the winter compared to the summer. In addition, it might be that variations between years, like the severity of the winter or prevalence of fever, influences the symptom burden of the children as well.

**Fig 4:**
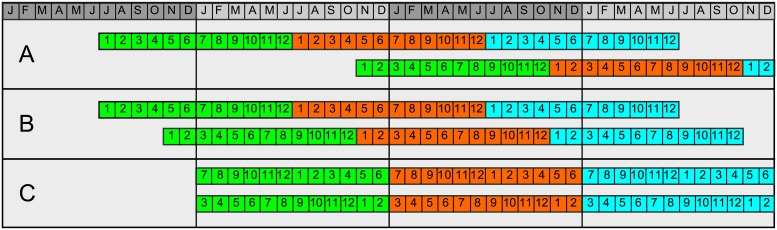
A schematic presentation of the alignment two diaries. Unaligned data (A), two diaries centered around January the first, neglecting year of birth (B) and centered and restructured samples neglecting year of birth and serial correlation of a single dataseries (C).

Fig 4 shows a schematic representation of two diaries measured at different points in time. The upper panel presents the two unaligned sequences. We assume that both data series have measurements for the complete three years. To keep the example simple, we have depicted the measurements as if taken at the level of months (instead of days for the real data). The first child enrolled the study in July of the first year, the second in November of year two.

Different strategies can be adopted to (partially) correct for the distortions listed above. A quick approach is to align both diaries based on the day of the year, as (schematically) depicted for one symptom in panel B of Fig 4. By aligning the data, for instance, around the first day of a calendar year, the age differences are kept to a minimum while seasonal effects are preserved. Effects on the level of years are neglected, but they can be included as a covariate in the analysis. In addition, this approach preserves the ordering of the time dimension of the individual signals and one could utilize the serial correlation in further analysis. A downside is that it leads to empty positions at the beginning and end of the time domain and not all statistical methods will be able to deal with that.

**Fig 5:**
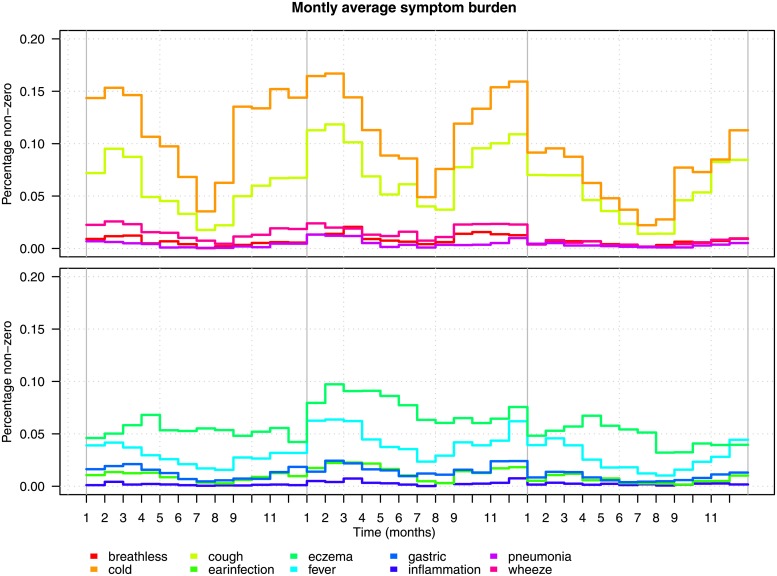
The monthly average scores for all symptoms calculated over all children.

This problem can be resolved by cutting the signals at the centering date (the first of January) and reposition the part in front of the cut at the same position, only twelve months later along the time axis. The data occupying these positions are moved to the same positions twelve months later in time, this process is repeated for the whole signal. The lower panel of Fig 4) (C) shows a graph of the result. The serial correlation of the data is lost, as is the information about the actual year when the measurements were taken. The advantage is that this type of restructuring leads to a matrix (or cube) without empty cells. Merging all matrices results in the *I* × *M* × *T* tensor $\underset{\bar{}}{X}$, with (*i* = 1, …, *I*) diaries or children, (*m* = 1, …, *M*) symptoms and (*t* = 1, …, *T*) points in time. Notice that we have chosen to summarize the signals on the levels of months, after centering, cutting and repositioning which makes *T* = 36. Fig 5 presents the monthly average scores for all symptoms calculated over all children, given this alignment. All symptoms are split over two plots just for clarity. The seasonal patterns are preserved and can be clearly observed in the patterns of cold and cough. The serial correlation between the years is lost and seems to cause some increased irregularities in the curves when jumping from December to January. Notice that the lines in the plot are averages and as such only serve as rough indication of the development of all symptoms over time.

#### Pattern based comparisons

One of the problems often occurring when analysing long signals is that it becomes increasingly difficult to correct for shifts and distortions in the time dimension (see e.g. [5] for a discussion). In the previous section we have tried to correct for distortions by reordering of the data and by summarizing the data on the level of months. This is clearly a compromise because although the seasonal effects are covered, (small) age differences do still exists.

An alternative, which bypasses discussions about optimal aligning strategies, is the ‘bag of patterns’ representation of data (see e.g. [6]). This technique is used in text mining to assess the degree of similarity between text documents. In such an analysis the words present in a text are the defining features, the overlap in the words (and their frequency) between documents is used as a measure of closeness. This idea is adapted for the analysis of signals by [7]. Long signals are translated into collections of symbols by discretizing both the intensities of the observed signals and the time dimension. In both applications the feature space is reduced to a table of counts. This table is used to search for repetitions of patterns within and across documents or signals. The location of these patterns are considered not important and are not a part of the model. Any reference to time or seasonality is thereby dropped.

In the present case we define the combination of symptoms on a given day as a single pattern. These patterns are basically codes consisting of zeros and ones. Three example patterns, highlighted by the grey bars, are given in Fig 1. The first pattern (A) yields the code 110, because the child coughs and suffers from a cold, but does not wheeze. The second pattern results in 010, because in that period the child only has a cold. Pattern (C) gives 101 for coughing and wheezing but no cold. Notice that this example is based on only three symptoms, while in our analysis we use ten. In addition, in the analysis described in this paper, patterns are derived on the level of a day, this instead of the wide intervals defined in Fig 1.

Vector *w* contains the collection of all (*g* = 1, …, *G*) observed patterns. Ten different symptoms would thus in theory lead to *G* = 2^10^, however, because some symptoms are very rarely occurring, only 363 unique patterns are observed. The pattern frequencies are summarized into matrix *F* for all (*j* = 1, …, *J*) diaries stratified over years. A small part of such a table is depicted in Table 1. For example, the first row contains all patterns observed for the first year of child one, which suffers 10 days of (only) coughing, 19 days of (only) wheezing and five days of the combination of coughing and cold. The second row, contains all patterns observed for the same child, only now for its second year of life. Assuming all diaries are completely filled-in, this table contains three rows for every child.

**Table 1:** Small example of a frequency table with patterns and diaries.

Child ID	Cough	Cold	Wheeze	…	Cough + Cold	…
001-y1	10	10	19	…	05	…
001-y2	11	05	05	…	02	…
001-y3	21	15	09	…	12	…
002-y1	17	01	13	…	16	…
002-y2	08	08	14	…	11	…

### Two-way data analysis

Table 2 present a number of exploratory methods applicable to the different representations of the data, those presented in boldface are explained below. The results of applying these methods to the data are presented in the next section. We distinguish between methods applicable to two-way data and three-way data. Notice that the table only shows a selection of the many methods which can be used for the clustering of symptoms. We start with a discussion of methods applicable to the matricised data, in a next step three-way decompositions are discussed.

**Table 2:** A number of possible methods that can be used given the specific representation of the raw data. Methods discussed in the text are printed in bold type.

Dimensions	Shape	Pattern
2-way	Heuristics	Heuristics
	**NMF**	(P)LSA
	Mixtures	**LDA**
3-way	PARAFAC	PARAFAC
	**INDSCAL**	INDSCAL
		LDA

Matricization of $\underset{\bar{}}{X}$ into a large matrix is very tempting since it enables the use of a wide range of easy accessible unsupervised algorithms (see [8] for a discussion on the translation of tensors to matrices). Here we investigate matricization over the features which means that each child is represented by one row in the obtained matrix. This yields matrix $\overset{ˇ}{X}$ of dimensions *I* × *MT*, each column of this matrix gives the burden of symptom *m* given time *t*. Notice that alternative ways of matricization of $\underset{\bar{}}{X}$ are possible, which opens alternative ways of looking at the data.

Matrix factorizations are highly successful in clustering tasks in practically every data domain. One of the advantages of these methods is that they allows us to reduce the feature space to a lower number of dimensions which makes the problem at hand more comprehensible. A prominent algorithm is the Singular Value Decomposition (SVD), which decomposes the data in three matrices. These matrices are subsequently used to investigate grouping structures in the samples and the variables. We can apply the SVD to the shape based matrices but also to the frequency matrix *F*. The latter case is known as Latent semantic analysis (LSA) [9] [10] in text analysis. However, problematic about the SVD is that the new feature space is in some applications difficult to interpret.

One alternative is non-negative matrix factorization (NMF) [11]. It decomposes $\overset{ˇ}{X}$ into two matrices:

$$\begin{array}{c} \overset{ˇ}{X}=UV^{\prime }, \end{array}$$ (1)

when $\overset{ˇ}{X}$ consists of *I* rows and *MT* columns, *U* is a *I* × *R* matrix and relates the samples with the new feature space, *V* is a *MT* × *R* matrix and represents the weights of the original features given the new components. Both matrices are non-negative which is a attractive property when studying data only containing positive values. In contrast to the SVD, NMF does not impose orthogonality on the *R* components. Instead of keeping all information in the reconstruction, one usually retains only a small number of components *R* < < *I*, resulting in a simplified or compressed representation of the data.

$$\begin{array}{c} \overset{ˇ}{X}\approx U_{R}V_{R}^{\prime }. \end{array}$$ (2)

Writing the NMF in the outer product form shows its close relation with the tensor decompositions which we will discuss later

$$\begin{array}{c} \overset{ˇ}{X}\approx \sum_{r=1}^{R}u_{r}v_{r}^{\prime }. \end{array}$$ (3)

Alternatively, one can employ model based clustering algorithms. The basic assumption of these models is that the data are generated according to a mixture distribution consisting of an (unknown) number of components or clusters. A latent variable *z* is introduced so that *z*_*k*_ represents the unobserved group to which the diary belongs. Most notable in this vein is the mixture model [12], which is adapted to a wide range of applications. A strongly related model, tailored to text analysis, is known as probabilistic latent semantic analysis (PLSA) [13].

Latent Dirichlet allocation (LDA) is a further extension of these models and is presented in [14] and is also known as a mixed membership model [15]. The idea of LDA is similar to mixtures and PLSA, but it takes a Bayesian approach instead of maximum likelihood estimation. Diaries are represented by a mixture of topics (clusters of variables), each topic is a latent multinomial variable characterized by a distribution over the (fixed) collection of patterns. Dirichlet priors are placed over the diary distributions over topics and on the topic distributions over patterns. The generative process of LDA can be described:

1. Draw distributions over patterns; *K* multinomials *β*_*k*_ from a Dirichlet prior *η*, one for each topic *k*
2. Draw vectors of topic proportions; *J* multinomials *θ*_*j*_ from a Dirichlet prior *α*, one for each diary *j*
3. Pick a topic *z* with probability *p*(*z*|*j*)
4. Generate a pattern *w* with probability *p*(*w*|*z*)

This corresponds to the joint distribution of the observed and latent variables,

$$\begin{array}{c} p\left(\beta ,\theta ,z,w\right)=\prod_{k=1}^{K}p\left(\beta_{k}\right)\prod_{j=1}^{J}p\left(\theta_{j}\right)[\prod_{g=1}^{G}p\left(z_{j,g}|\theta_{j}\right)p\left(w_{j,g}|\beta_{1:K},z_{j,g}\right)]. \end{array}$$ (4)

In practice this process needs to be inverted, which amounts to estimating the posterior distribution, i.e. the conditional distribution of the topic structure given the observed data:

$$\begin{array}{c} p\left(\beta ,\theta ,z|w\right)=\frac{p(\beta ,\theta ,z,w)}{p(w)}. \end{array}$$ (5)

Exact inference of this distribution is intractable, two types of approximation algorithms are used in the literature. Variational methods [14] and sampling based algorithms [16].

### Three-way data analysis

To preserve the three dimensions of the data so-called multi-way methods can be applied. These methods are popular in chemometrics and psychometrics, for overviews in both areas we refer to [17] and [18]. Here we consider individual differences scaling (INDSCAL), which is a special case of parallel factor analysis (PARAFAC) [19] [20]. PARAFAC is a decomposition method that generalizes the bilinear principal component analysis (PCA) to multi-way data. An *R* component PARAFAC model of $\underset{\bar{}}{X}$ can be written as:

$$\begin{array}{c} x_{itm}=\sum_{r=1}^{R}a_{ir}b_{tr}c_{mr}+e_{itm}. \end{array}$$ (6)

Where PARAFAC is loosely defined as the multi-way alternative to PCA, INDSCAL is considered the higher order alternative to Multi Dimensional Scaling (MDS). In MDS a model is created for a single *I* × *I* matrix with dissimilarities. INDSCAL analyses a series of dissimilarity matrices in one model. We calculate point wise differences, meaning differences given symptom and time, between the samples in the tensor $\underset{\bar{}}{X}$, which results in $\underset{\bar{}}{D}$ with dimensions *I* × *I* × *TM*. INDSCAL is effectively a PARAFAC model with the restriction that coefficients matrix *B* = *A*:

$$\begin{array}{c} x_{ii^{\prime }l}=\sum_{r=1}^{R}a_{ir}a_{i^{\prime }r}c_{lr}+e_{ii^{\prime }l}, \end{array}$$ (7)

with *l* = 1, …, *TM*. Components derived with PARAFAC or INDSCAL are not orthogonal, as opposed to PCA. Estimation of PARAFAC and INDSCAL models is done in an iterative manner and the solutions are unique under mild assumptions. There are a number of suitable algorithms to perform this task, very often alternating least squares is used (see e.g. [21]). In the present models the only parameter that needs to be optimized is the number of components. Here we rely on heuristics, although a number of alternatives are available. Some examples are cross-validation and residual plotting, more methods and references can be found in [17].

## Results

This section presents the results of the different analyses. We distinguish between results based on the shape based representation and the pattern based representation. All analysis are performed in R [22], except for the INDSCAL model which if fitted using the N-way toolbox [23] available for Matlab.

### Shape based analysis

As a first analysis, NMF is applied to matrix $\overset{ˇ}{X}$. Eight models are fitted, with a rank one solution for the first up to rank eight for the last model. Because the NMF can end up in local minima, each model is initiated 25 times after which the best fitting solution is stored.

**Fig 6:**
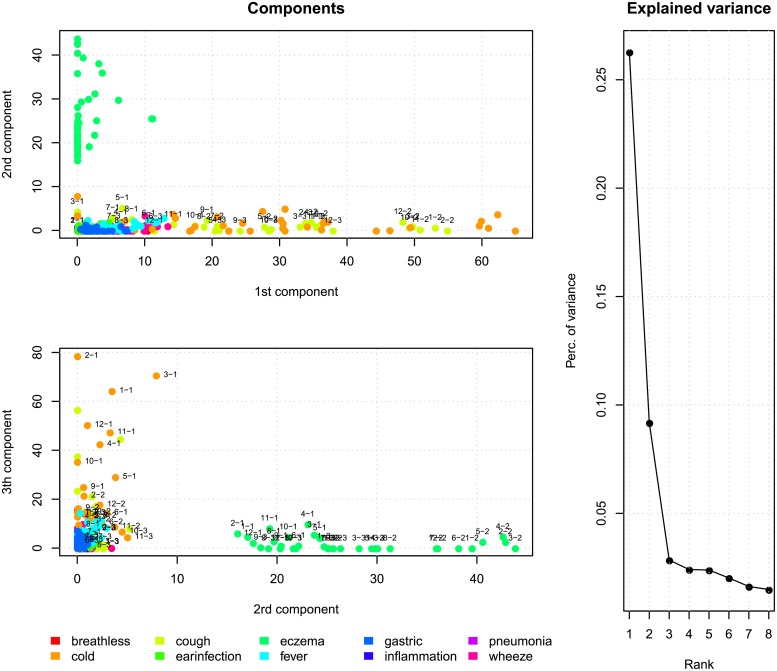
Results of a three component NMF applied to the matrix $\overset{ˇ}{X}$.

The results of this analysis are presented in Fig 6. The right panel shows the additional explained variance in the data using the first *r* components, compared to the model with *r* − 1 components. Because the components are not orthogonal, this figure should be interpreted in terms of models and not in terms of the additional contribution of single components. The figure shows that after three components the additional explained variance levels off. Using this plot to decide on the number of components, similarly as the scree plot is used in factor analysis, one is most inclined to choose three components. The three component model explains 38 percent of the variance, adding a fourth or fifth component will explain cumulatively 40 and 43 percent of variance respectively.

The two panels on the left of Fig 6 show the association between the input variables and the three estimated components, given the three component solution. Every dot in both figures is a symptom, month—year combination. Some symptoms are labelled (month—year) in order to unveil possible trends in time. The first component defines the symptoms cold and cough, since these are the only symptom with substantial weights in this direction. The second component is dominated by eczema, the third mainly by cold and also cough has substantial loadings in this component. Especially eczema behaves relatively independent from the other symptoms. Increasing the number of dimensions from three to four of five leads to components in which cough and cold are important, eczema, however, remains in a separate dimension.

**Fig 7:**
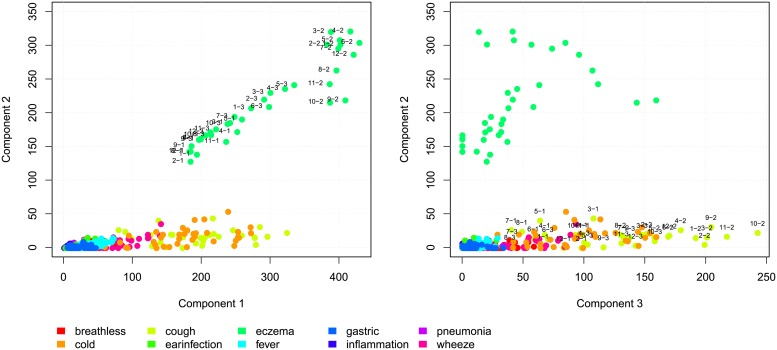
The components, resulting from the INDSCAL model, plotted against each other.

To utilize the three-way structure of the data we have also applied INDSCAL. The N-way toolbox allows a number of constraints, here we apply a non-negativity constraint on the symptom mode. We choose a three component model, the results are plotted in the two panels of Fig 7, the dots are (color) coded in the same way as in Fig 6. The left panel shows component one versus component two. The first component can be interpret as the general prevalence of the different symptoms. Eczema, coughing and cold are the most abundant symptoms, while for instance pneumonia is rare. Components two and three are more informative in terms of single or related groups of symptoms. Component two is discriminative for eczema, the third component separates cough and cold from the other symptoms. The INDSCAL model with three components explains 40.2 percentage of variance in the data. When choosing a four component model, 42.5 percent is explained.

### Pattern based analysis

For the pattern based analysis we rely on the LDA model (using the Topicmodels package in R [24]). After experimenting with three to six topics and compare the results, we fix the the number of topics to be four. For more formal optimization strategies, as used in text mining, we refer to [25]. The parameters of the model are estimated using a Gibbs sampler, the hyperparameters are set to the default values *η* = 0.1 and *α* = 50/*K* (see [16] for discussion) and are updated in the Markov chain. The chain consisted of 10000 iterations, with a burnin period of 2000 iterations.

After estimation all patterns (symptoms or symptom combinations) are connected to the topics by a certain weight. For each topic the top five of most relevant patterns are presented in Table 3. The LDA model provides weights which are directly related to the per term distribution over topics. These weights are printed in Table 3 for every term and express the importance of that term within a particular topic.

**Table 3:** Top five terms for each of the four topics found by LDA.

Topic 1	Topic 2	Topic 3	Topic 4
Eczema (0.77)	Cough cold (0.46)	Cold (0.97)	Fever (0.30)
Cold eczema (0.08)	Cough cold fever (0.09)	Cold fever (0.02)	Gastric (0.19)
Cough eczema (0.03)	Cough wheeze cold (0.05)	Cold gastric (<0.01)	Cold fever (0.16)
Cough cold eczema (0.03)	Cough wheeze (0.05)	Cold fever gastric (<0.01)	Earinfection (0.10)
Fever eczema (0.01)	Wheeze (0.04)	Wheeze cold earinfection (<0.01)	Fever gastric (0.05)

From the results we observe one topic which strongly focuses on eczema symptoms, a topic topic which forms a combination of cough a cold symptoms and a third topic which is dominated by cold symptoms. In the fourth topic fever is the most important symptom, nevertheless it is a fairly mixed topic since also other (combinations of) symptoms receive considerable weight.

## Discussion and conclusions

Central to this paper are diary data, collected to investigate the asthmatic related symptoms burden of young children. The characteristics of the data are relative unusual, as such we have focused on the methodological aspects of analysing these data. We discern two, related, methodological issues. First there is the problem of processing the (binary) diaries in such a way that they are suitable for analysis. Two types of data representations are discussed, the shaped based representation and pattern based representation. The second problem follows from the first and concerns which statistical tools are the most applicable given the data representation.

The shape based representation requires the data to be aligned, after which they can be compared on a point by point basis. Proper alignment is however delicate, but when feasible many tools are applicable. The big advantage of this approach that (detailed) information in the time dimension is preserved and hypotheses involving time effects can be tested. Summarizing the data, say on the level of months, reduces the alignment problems but comes with a loss of resolution. For the shape based analysis we have applied NMF to the matricised data, and used INDSCAL for the three-way data. The data are summarized as monthly proportions; the number of days a certain symptom is present. For both NMF and INDSCAL we choose a three component model. The components derived from the models are relative similar in terms of their interpretation, INDSCAL is able to explain slightly more variance. In the present case there is still a moderate amount of structure in the time dimension, largely due to the seasonal effects. This effect seems to be captured by both the NMF and INDSCAL since the results of both methods pickup seasonal effects.

When symptom episodes are occurring more at random locations in time, a point by point comparison becomes less applicable and shape based approaches will fail to come up with satisfactory results. In such cases, pattern based methods will be of much greater use. The pattern based representation focuses on reoccurring patterns and ignores ordering in time. With this representation we stratify the data on the level of years, so that possibly yearly differences can still be detected. A further stratification of the data, from years to e.g. seasons, is possible. However, due to the sparseness it might be that a further reduction of the period length makes the estimates unstable. The data are analysed using Latent Dirichlet allocation, a method popular in text mining applications. The application of LDA to this data is an interesting experiment since it it usually applied in situations with much more data available, still it derives sensible topics. The results coming from the LDA are well interpretable and seems to deliver relative similar (conceptual) dimensions as the shape based method do.

Both methods are in relative agreement with respect to which symptoms are deemed important in the data. As such it seems that the grouping of the features, basically along the lines of cold, cough and eczema are robust. At the same time we must conclude that these are the dominant symptoms in terms of the raw counts and the methods seem to be less capable of detecting clear patterns in the less abundant symptoms. In a future study, one of the aims should be to zoom in on these rare occurring symptoms and see whether not their frequency but maybe their timing or co-occurrence with other symptoms can be related to specific characteristics of the children. In addition, it would be interesting to test both representation in a supervised setting and accommodate the inclusion of additional covariates.
